# Prosthetic Aortic Valve Infective Endocarditis Complicated With Malignant Arrhythmias: Diagnostic and Management Challenges

**DOI:** 10.7759/cureus.118028

**Published:** 2026-10-16

**Authors:** Imthiaz Manoly, Safia Fathima Anver Pasha, Mohannad M Al Asaad, Nayyer Siddiqi

**Affiliations:** 1 Cardiothoracic Surgery, Dubai Hospital, Dubai Health, Dubai, ARE

**Keywords:** aortic root abscess, ecmo, infective endocarditis, malignant arrhythmia, prosthetic valve endocarditis, redo sternotomy

## Abstract

Prosthetic valve endocarditis (PVE) remains a serious, life-threatening complication of cardiac valve replacement, with outcomes significantly determined by timely recognition, multidisciplinary management, and appropriate surgical intervention. We report the case of a 59-year-old man with a prior mechanical aortic valve replacement, previously treated for iron deficiency anemia with recommended further workup but lost to follow-up, who presented with chest pain, hemodynamic compromise, and cardiac arrhythmias. Echocardiography confirmed severe prosthetic valve dehiscence with a paravalvular leak and peri-aortic root abscess consistent with PVE; blood cultures grew *Staphylococcus epidermidis*. His course was complicated by cardiogenic shock secondary to severe aortic regurgitation and bradyarrhythmias requiring emergency transvenous pacing. Despite an urgent redo aortic valve replacement with aortic root reconstruction and perioperative veno-arterial extracorporeal membrane oxygenation (VA-ECMO) support, he subsequently developed refractory malignant arrhythmias and could not be resuscitated despite extensive efforts.

This case highlights the diagnostic challenges of PVE presenting with nonspecific features, the surgical complexity of valve replacement in the setting of advanced aortic root and periannular destruction, and the catastrophic potential of postoperative malignant arrhythmias following complex infective endocarditis surgery. It underscores the critical importance of early recognition, dedicated endocarditis teams, and the need for further research into the management of postoperative arrhythmias in this high-risk population.

## Introduction

Infective endocarditis (IE) is a rare but life-threatening infection affecting the endothelial lining of the heart, most commonly involving native or prosthetic cardiac valves and, less frequently, other implantable intracardiac structures or devices. The global annual incidence of IE is estimated at approximately 3-10 cases per 100,000 person-years [1]. Predisposing factors for IE include congenital cardiac anomalies, a prior history of IE, intravenous drug abuse, indwelling cardiac devices, the type of microorganism, including fungi, and immunosuppression [2].

Prosthetic valve endocarditis (PVE) is a serious complication of valve replacement surgery, defined as an infection involving a surgically or transcatheter-implanted cardiac valve substitute, with an overall incidence of 0.3-1.2 cases per 100 patient-years, accounting for 10-30% of all IE cases [3]. The risk of PVE can be influenced by valve type, with bioprosthetic valves exhibiting a higher risk for developing PVE as compared to mechanical valves during long-term follow-up; however, comparative evidence remains inconsistent across distinct cohorts [4,5]. Despite advances in early diagnosis, antimicrobial therapy, and surgical management, PVE remains associated with significant morbidity and mortality, with in-hospital mortality rates ranging from 20% to 30%, with even higher rates in the presence of aortic root abscess, conduction abnormalities, or haemodynamic instability [6].

Following surgical intervention for infective endocarditis, there is an elevated risk of developing postoperative arrhythmias which may complicate recovery. These may range from transient self-limited rhythm disturbances to life-threatening tachyarrhythmias or bradyarrhythmias, requiring urgent pharmacological or interventional management. Despite advances in perioperative and electrophysiological care, postoperative malignant arrhythmias refractory to conventional management remain a complex and potentially catastrophic complication following IE surgery [7].

This case report highlights the atypical presentation of PVE in a patient with a mechanical aortic valve, the diagnostic challenges of the disease, and the multidisciplinary management of advanced annular destruction requiring redo sternotomy, root reconstruction, perioperative VA-ECMO support, and postoperative malignant arrhythmias.

## Case presentation

A 59-year-old man presented to the emergency department with a three-hour history of left-sided chest pain radiating to the left shoulder, partially relieved by rest, accompanied by three episodes of non-bilious vomiting and dizziness. Vital signs were initially within normal limits except for mild tachycardia. On further history, he acknowledged feeling generally unwell for the preceding two months without seeking medical attention.

His past medical history was significant for hypertension, managed with a beta-blocker and an angiotensin receptor blocker, and mechanical aortic valve replacement performed four years prior for aortic valve stenosis in his home country. He was maintained on lifelong warfarin and aspirin. He was a non-smoker with no history of alcohol or illicit drug use.

Prior admission: initial presentation

Approximately five months before the current presentation, the patient was admitted to our institution with a 10-day history of mild hemoptysis, dizziness, dyspnea, and exertional chest pain. He was hemodynamically stable, and investigations at that time revealed symptomatic microcytic anemia likely secondary to iron deficiency with elevated N-terminal pro-B-type natriuretic peptide (NT-proBNP).

A CT scan of the chest with aortogram showed no aortic aneurysm or dissection, minimal pleural effusion, patchy upper-zone pulmonary infiltrates, and intact sternotomy wires, as illustrated in Figure 1. 

**Figure 1:**
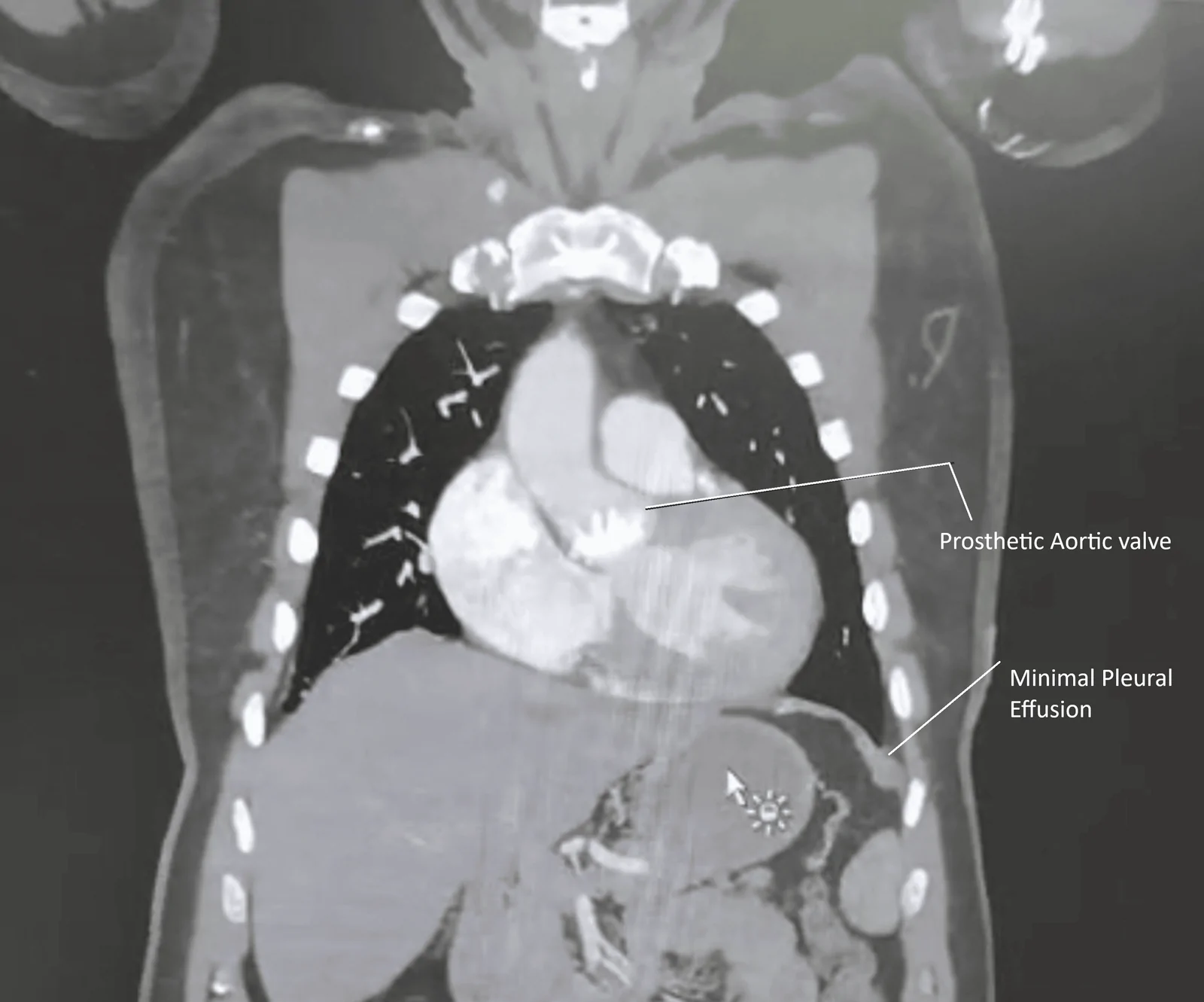
Coronal section of the CT aortogram demonstrating the prosthetic aortic valve and minimal pleural effusion on initial presentation. There were no other significant findings.

He was treated symptomatically with packed red blood cell transfusion, intravenous iron, and therapeutic low-molecular-weight heparin. He was referred for further investigations of his anemia, including bronchoscopy, upper and lower gastrointestinal endoscopy. As the patient improved symptomatically with near correction of his anemia, he was discharged with a plan to investigate this condition further as an outpatient. The patient did not attend his follow-up investigations. The transthoracic echocardiogram (TTE) performed during his previous admission was reviewed during the current admission and demonstrated a mild paravalvular leak of the aortic valve. In retrospect, this finding, along with his clinical presentation, raises the possibility of an evolving prosthetic valve infection which could have caused slight dehiscence; however, clinical and microbiological evidence were insufficient to establish a diagnosis of PVE at that time.

Current admission: investigations and clinical deterioration

During his current visit, physical examination demonstrated progressive hemodynamic instability. Cardiac auscultation revealed a diastolic murmur with a metallic valve click, while bilateral basal crepitations were auscultated on pulmonary examination. He subsequently developed recurrent episodes of profound bradycardia leading to complete heart block, which was suggestive of perivalvular extension of the infection. Due to an inability to maintain an adequate mean arterial pressure (MAP), he was initiated on vasopressor support with noradrenaline and dopamine, and atropine was administered for persistent bradycardia. In view of his bradyarrhythmia, a temporary transvenous pacemaker was inserted and programmed at 70 bpm (beats per minute), and later adjusted to 60 bpm (Figure 2). The lab investigation timeline can be seen in Tables 1-4. 

**Figure 2:**
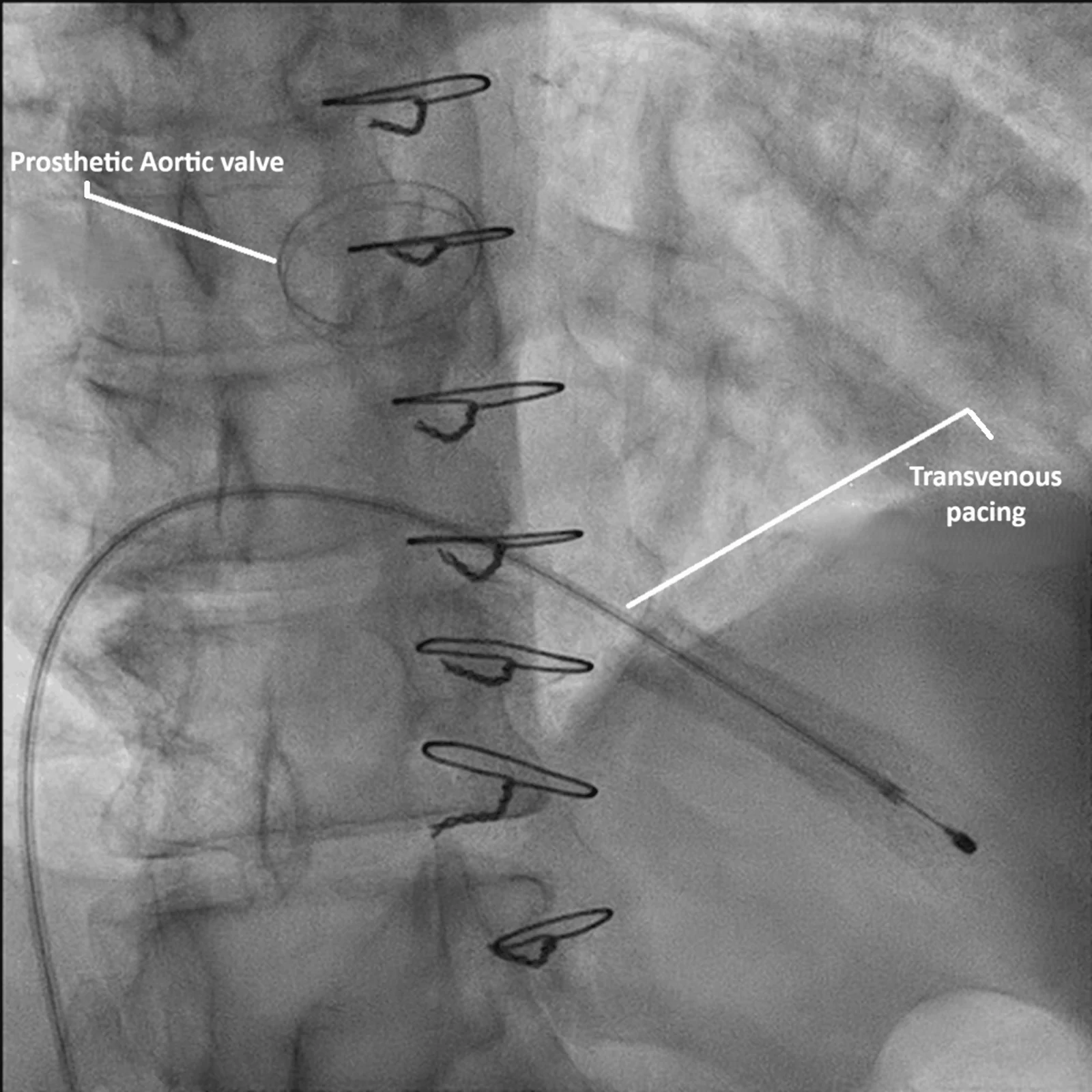
Transvenous pacing insertion. Note the sternal wires, prosthetic aortic valve and the pacing cable inserted through the femoral vein and the inferior vena cava.

**Table 1:** Iron studies from the initial presentation

Lab parameter	Reference range	Initial presentation
Iron (Fe)	59-158 µg/dL	16
Transferrin	2.12-3.60 g/L	3.28
TIBC	250-435 ug/dL	417
Iron saturation	15-45%	4
Ferritin	30-400 ng/mL	20.9

**Table 2:** Complete blood count (CBC) and inflammatory markers Note the significant rise in inflammatory markers, including WBC count, CRP, and PCT, in a short span of time. WBC: white blood cells; RBC: red blood cells; RDW: red cell distribution width; MCV: mean corpuscular volume; MCH: mean corpuscular hemoglobin.

Lab parameter	Reference range	Initial presentation	Day 1	Day 4	Day 5
WBC count	3.6-11.0 x 10 ^3^/µL	5.2	14.7	26	42.4
RBC count	4.50-5.50 10 ^6^/µL	3.69	4.72	4.03	3.65
Hemoglobin (Hb)	13-17 g/dL	6.9	10	10	9.0
Hematocrit (Hct)	40-50%	23.1	31.3	31.4	27.8
MCV	77-95 fL	62.7	66.3	77.8	76.3
MCH	27-32 pg	29.6	21.1	32	32.4
RDW	11.5-14%	20.2	20.1	23.6	24
Platelet count	150-410 x 10^3^/µL	321	213	156	153
C-Reactive Protein (CRP)	<5 mg/L	8.3	173	337	337
Procalcitonin (PCT)	<0.05 ng/mL	0.05	0.28	30.90	30.90
Lactate	0.5-1.6 mmol/L	1.9	3.5	16	3.6

**Table 3:** Renal function tests (RFTs) and serum electrolyte abnormalities Note the significant derangement of the renal function as evidenced by the rise in urea and creatinine, and fall in eGFR. eGFR: estimated glomerular filtration rate.

Lab parameter	Reference range	Initial presentation	Day 2	Day 4	Day 5
Creatinine (Cr)	0.70-1.20 mg/dL	1.07	1.21	2.14	3.29
eGFR	> 60 mL/min/1.73 m^2^	80.4	69	34.8	20.8
Urea	12-40 mg/dL	31	41	100	115
Sodium	136-145 mmol/L	145	129	147	148
Potassium	3.4-4.5 mmol/L	4.8	4.6	3.8	4.4
Chloride	98-108 mmol/L	108	97	107	114
Bicarbonate (HCO3)	20-28 mmol/L	26	20.7	14.1	22.7

**Table 4:** Cardiac markers and coagulation profile Note the significant rise in NT-proBNP levels, which is indicative of heart failure. PT: prothrombin time; aPTT: activated partial thromboplastin time; INR: international normalized ratio; NT-proBNP: N-terminal pro–B-type natriuretic peptide; CK-MB: creatine kinase-MB.

Lab parameter	Reference range	Initial presentation	Day 2
Troponin T	<14 ng/L	19	57
CK-MB	<6.23 ng/mL	1.9	--
NT-proBNP	<125 pg/mL	2683	19,643
PT	12.6-16.3 secs	18.1	40.6
INR	0.8-1.1	1.36	2.43
aPTT	25.1-37.7 secs	39.8	52.3

His baseline ECG demonstrated a left bundle branch block with a junctional rhythm and AV dissociation. Subsequent tracings revealed atrial fibrillation, broad R waves in leads I, V5, and V6, prolonged QRS duration, and persistent left bundle branch block, findings consistent with progressive conduction system involvement (Figure 3)**. **

**Figure 3:**
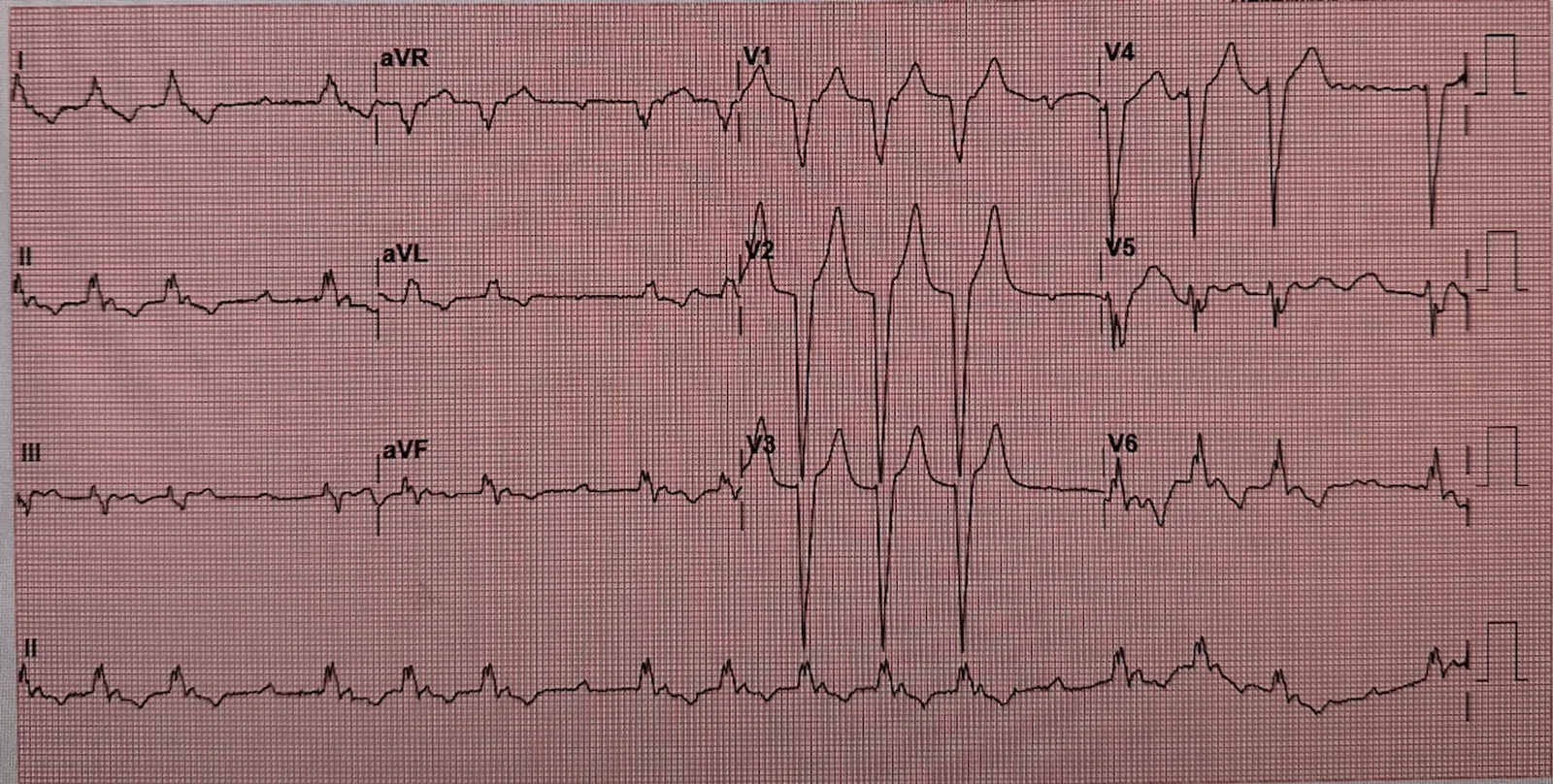
Baseline ECG illustrates a left bundle branch block with prolonged QRS and broad R waves in the left lateral leads

The TTE demonstrated severe aortic valve dehiscence causing rocking valve movement, with severe paravalvular leak and aortic regurgitation (Video 1). Thickening of the aortic root was also noted, concerning for a peri-aortic abscess. There was mild impairment of left ventricular systolic function. His initial left ventricular ejection fraction was 60-65% and dropped to 35% as his condition worsened. Additional findings included severe concentric left ventricular hypertrophy, grade II left ventricular diastolic dysfunction, severe left atrial dilatation, moderate mitral regurgitation, and mild tricuspid regurgitation. Subsequent trans-oesophageal echocardiography (Video 2) demonstrated a large aortic root abscess, measuring 1.5 cm in thickness and extending from the 7 to 2 o’clock positions in the short-axis view, with an associated pseudoaneurysm within the abscess, in addition to the TTE findings described above.

**Video 1:** Parasternal long-axis view of the transthoracic echocardiogram demonstrating rocking motion of a dehisced prosthetic aortic valve with vegetation

**Video 2:** Transesophageal echocardiogram demonstrating the dehisced prosthetic aortic valve with aortic root abscess extending into the aortomitral intervalvular fibrosa

Three sets of blood cultures were collected prior to initiation of antibiotic therapy. Although no growth was detected in the first 24 hours, growth of Gram-positive cocci in clusters was observed later. All three sets ultimately grew methicillin-resistant *Staphylococcus epidermidis*independently, reflective of true bacteraemia in the context of infective endocarditis. Urine and wound cultures returned negative. Empirical therapy with vancomycin and ceftriaxone was commenced. Following infectious diseases consultation, the antimicrobial regimen was escalated to vancomycin, cefepime, and gentamicin in accordance with the established guidelines for PVE.

The case was discussed in the heart team meeting, which concluded with the establishment of the PVE diagnosis [8] with hemodynamic instability.

Surgical management

Given the severity of valve dehiscence, hemodynamic compromise, and established aortic root abscess, the patient was taken urgently to the operating theatre for redo sternotomy despite the very high operative risk.

A redo sternotomy was performed after exposing the right femoral vessels. Cardiopulmonary bypass was established via right femoro-right atrial cannulation. The patient was cooled down to 28°C. A U-shaped aortotomy was fashioned, and the infected prosthetic valve with large vegetations was explanted and sent for microbiological and histopathological analysis (Figure 4). The aortic root abscess extended in two directions: first, between the non-coronary and left coronary cusps to the anterior leaflet of the mitral valve, and second, beneath the non-coronary cusp of the aortic valve, deep toward the right atrium. Both cavities were incised, extensively debrided, and then reconstructed with bovine pericardial patches, using a modification of the hemi-Commando approach, as described in the literature [9].

**Figure 4:**
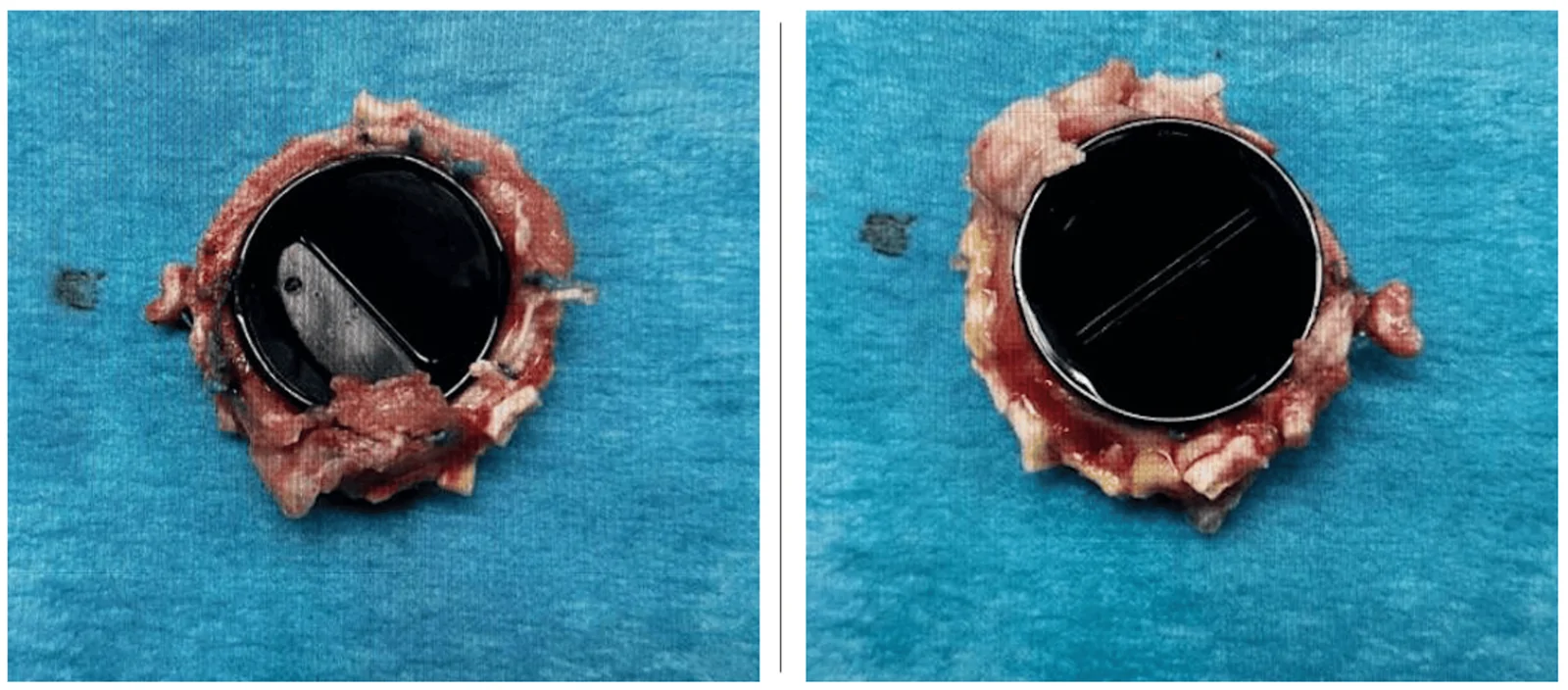
Intraoperative images demonstrating the explanted prosthetic aortic valve with vegetation and part of the aortic root abscess cavity Note the excised abscess cavity wall with a chunk of ventricular wall in the 6 o'clock position which corresponded to the area beneath the non-coronary cusps.

The anterior mitral valve leaflet was repaired with a bovine pericardial patch. A 21 mm Carpentier-Edwards PERIMOUNT Magna Ease bioprosthesis (Edwards Lifesciences Corp., Irvine, CA) was implanted in a supra-annular position with interrupted 2-0 pledgeted sutures. The epicardial pacing leads (two atrial, two ventricular) were initially secured, and VVI pacing was initiated at a rate of 90 bpm. VA-ECMO was established intraoperatively given significant myocardial injury and anticipated hemodynamic instability.

Postoperative course

The patient was transferred to the surgical intensive care unit on VA-ECMO (FiO₂ 80%, sweep 3 L/min, 3,000 rpm) and high-dose inotropic support (noradrenaline, adrenaline, and milrinone). In view of his rising septic markers and labile blood pressures, he progressed to septic and hypovolemic shock. Furosemide infusion was commenced for oliguria, and systemic anticoagulation was maintained as per the institutional ECMO protocol. Pleural and mediastinal drains were in situ. Continuous veno-venous haemodialysis was initiated for oliguria, hyperkalemia, metabolic acidosis, and acute kidney injury.

The patient then developed recurrent malignant ventricular arrhythmias, requiring multiple rounds of unsynchronized and synchronized direct current cardioversion. The arrhythmia began as non-sustained ventricular tachycardia (NSVT) and was initially managed with correction of electrolyte imbalances, including magnesium and potassium infusions. He then developed atrial fibrillation, followed by premature ventricular complexes, and finally progressed to sustained ventricular tachycardia (VT). A lidocaine infusion was commenced after multidisciplinary input from cardiology, intensive care, and electrophysiology. He went on to develop arrhythmias of various morphologies over a short period of time that were resistant to standard treatment. VT ablation was discussed but could not be performed given the pace of clinical deterioration.

Following a turbulent postoperative course lasting over three days, the patient suffered a cardiac arrest on the final day. Advanced cardiac life support with ECMO was initiated; however, resuscitative efforts were unsuccessful, and the patient was pronounced deceased.

## Discussion

Diagnostic challenges in PVE

PVE poses significant diagnostic challenges due to its heterogeneous presentation and the limitations of conventional diagnostic criteria. The Modified Duke Criteria, now augmented by advanced imaging modalities including 18F-FDG PET/CT and radiolabelled white cell scintigraphy, have improved diagnostic sensitivity, particularly in prosthetic valve disease where echocardiography may be limited by acoustic shadowing and artefacts [8].

Culture-negative endocarditis occurs in up to 31% of IE cases and is frequently attributable to prior antibiotic administration, fastidious, intracellular organisms (*Haemophilus, Aggregatibacter, Cardiobacterium, Eikenella, Kingella* (HACEK); *Coxiella burnetii*, *Bartonella* spp., *Tropheryma whipplei*), or fungal pathogens. A systematic approach including repeated blood cultures, extended incubation, serology, PCR of valve tissue, and 16S rRNA sequencing should be pursued in culture-negative cases [10,11].

This patient's prior admission five months earlier, characterised by anemia, elevated cardiac biomarkers, and pleuropulmonary changes, in hindsight, may have represented an unrecognised earlier phase of IE that was incompletely investigated. This highlights the importance of maintaining a high index of suspicion for IE in patients with prosthetic valves presenting with unexplained systemic illness.

Surgical timing and operative complexity

The optimal timing of surgery in IE remains an area of active debate. The current European Society of Cardiology (ESC) guidelines for management of endocarditis recommend urgent surgery (within 3-5 days) for IE complicated by heart failure, uncontrolled infection, or high embolic risk. In prosthetic valve endocarditis complicated by aortic root abscess and haemodynamic compromise, as in this case, urgent surgery is generally indicated despite the elevated operative risk [12].

Redo cardiac surgery in the setting of active infective endocarditis carries substantially higher morbidity and mortality than index procedures, compounded by annular friability, tissue destruction, and the risk of reinfection. Aortic root abscess involving the aortomitral continuity is among the most surgically demanding scenarios, often requiring complex reconstruction techniques such as Commando or hemi-Commando procedures. The Commando procedure, which involves aortic root and mitral valve replacement with reconstruction of the aortomitral intervalvular fibrosa, is performed in extensive and destructive cases of infective endocarditis that are otherwise not amenable to conventional medical or surgical management. Despite the technical demands, these procedures have been associated with reasonably positive postoperative outcomes, including lower rates of re-intervention and favourable survival when performed by experienced surgical teams [13].

On the contrary, the modified hemi-Commando procedure, which combines aortic root replacement with reconstruction of the anterior mitral leaflet and aortomitral intervalvular fibrosa using pericardial tissue, represents an alternative approach to annular destruction of this extent with highly favourable outcomes. The reported benefits, such as reduced early postoperative mortality and improved long-term survival rates, may be attributed to the preserved posterior mitral valve leaflet and annulus, hence maintaining overall left ventricular function [14].

ECMO as a bridge in high-risk IE surgery

Mechanical circulatory support, particularly VA-ECMO, is increasingly recognised as a valuable adjunct in high-risk cardiac surgery. In the context of IE with severe myocardial dysfunction and anticipated postoperative instability, a perioperative decision can be made for the institution of ECMO, which can provide hemodynamic support while allowing the myocardium time to recover. However, ECMO in the setting of active infection and postoperative coagulopathy carries considerable risk, including bleeding, thromboembolism, and circuit-related complications [15].

Malignant arrhythmias in postoperative IE

Atrial fibrillation (AF) is the most common postoperative arrhythmia after cardiac surgery, with reported incidences of 20-30% following coronary artery bypass grafting (CABG) to 40-50% after valvular surgery [16].

Though uncommon, postoperative ventricular arrhythmias (POVA) such as premature ventricular contractions (PVCs) and ventricular tachyarrhythmias (ventricular fibrillation or tachycardia) occur with a reported incidence of ~0.7-1.4%. Non-sustained ventricular tachycardias (NSVT) and PVCs do not require acute treatment unless reversible causes of arrhythmias are found and treated. Whereas in sustained ventricular arrhythmias, pharmacological methods (antiarrhythmics such as lidocaine, procainamide, or amiodarone) or electrical cardioversion strategies (defibrillation, implantable cardioverter-defibrillators) are considered. POVA following surgery for IE is associated with high mortality and may reflect ongoing myocardial inflammation, ischaemia, electrolyte disturbance, or direct conduction system involvement by the infective process [17]. 

Postoperative bradyarrhythmias such as atrioventricular blocks can be anticipated in IE and can necessitate permanent pacemaker (PPM) implantation. The underlying mechanism of these electrical disturbances is likely septic destruction of the aortomitral curtain, which may be compounded during IE surgery by manipulation of tissue near or involving the conduction system [18]. 

In our patient, the extent of annular destruction and conduction system involvement, evidenced preoperatively by complete heart block, and an extensive surgery with debridement of the abscess cavity likely contributed to the development of refractory ventricular arrhythmias. Pharmacological therapy and cardioversion were administered while other potential strategies such as overdrive pacing and catheter ablation were considered; however, the rapidity of deterioration precluded any definitive intervention.

Hence, this case highlights an important gap in the current literature regarding the management of refractory postoperative arrhythmias in the setting of complex IE surgery. 

The role of the endocarditis team

International guidelines strongly advocate for the establishment of dedicated multidisciplinary endocarditis teams, comprising cardiologists, cardiac surgeons, infectious disease specialists, microbiologists, and imaging specialists [8]. Evidence suggests that care delivered by such teams is associated with improved adherence to guidelines and better clinical outcomes. This case illustrates how the complexity of IE spanning diagnosis, antimicrobial management, surgical timing, perioperative support, and arrhythmia management necessitates coordinated and multidisciplinary expertise at every stage.

## Conclusions

This case report highlights the challenges encountered in a patient with prosthetic valve endocarditis complicated by aortic root abscess with annular destruction and refractory postoperative malignant arrhythmias. It illustrates the cumulative impact of diagnostic delay, the surgical complexity of advanced PVE, and the limitations of current strategies for managing life-threatening postoperative arrhythmias in this setting. Prospective registry data and collaborative research are needed to further refine the management of these highest-risk patients, particularly concerning the prevention and treatment of arrhythmias following complex endocarditis surgery.
